# Diuretic Properties and Chemical Constituent Studies on* Stauntonia brachyanthera*

**DOI:** 10.1155/2015/432419

**Published:** 2015-12-29

**Authors:** Xuan Li Liu, Dan Dan Wang, Zi Hao Wang, Da Li Meng

**Affiliations:** School of Traditional Chinese Materia Medica, Key Laboratory of Structure-Based Drug Design and Discovery (Shenyang Pharmaceutical University), Ministry of Education, Wenhua Road 103, Shenyang 110016, China

## Abstract

The pharmacological evaluation demonstrated that the extracts from the stem of* S. brachyanthera* could significantly increase the outputs of urine of rats compared to those of furosemide treated group, and the effect could last for a longer period of time. The best effect appeared in the first two hours, which scientifically confirmed the diuretic effect of the plant. The comparative pharmacognosy study showed that the characters of the crude drugs of the stem of* S. brachyanthera* were similar to those of* Akebia caulis*. Further systemic work on its chemical constituents by chromatographic methods and NMR elucidations led to the isolation of 10 triterpenoids, 6 flavonoids, 4 lignanoids, and 3 phenylethanoid glycosides, whose structural types were much similar to those of* A. quinata*. Among them, 7 compounds were firstly reported in the genus of* Stauntonia* and calceolarioside B was the common characteristic constituent in both plants. From the similar pharmacognosy characters, pharmacological effects, and chemical constituents, it could be concluded that* S. brachyanthera* have a great possibility to be a succedaneum of* Akebia caulis*, whose supply is extremely short in recent years.

## 1. Introduction

Recent studies demonstrated that the impaired renal function, hypertension, hyperkalemia, and the side-effect of immunosuppressant might lead to severe disruption of the water homeostasis, and the retention of urine flow is crucial compensatory mechanisms of water retention [1–4]. The treatments of some diseases such as ascites of liver, heart failure, edema, and glaucoma also need intakes of diuretics [5, 6]. With an aim to resist the retention of urine caused by the above factors and to find effective medicines to treat diseases mentioned above, an effective and safe diuretic agent is very necessary.

Although so many synthetic medicines were used for this purpose, natural resources medicines are still an important choice because of their higher efficiency and better safety. As we all know that China is famous for its extensive utilization of herbal medicines, among which* Akebia caulis*, a traditional Chinese medicine called Mutong by Chinese people, has been proved for its distinguished effects of diuresis, tranquilization, and promoting menstruation [7, 8] and used for thousands of years as analgesics, antiphlogistics, and diuretics to treat the diseases such as edema, stranguria, and amenorrhea [9–11]. In Chinese Pharmacopeia [12],* Akebia caulis* used to have two main resources,* Akebia quinata* (Thunb.) Decne and* Aristolochia manshuriensis* Kom. However, with the discovery of the harm of aristolochic acid, which could induce the injury of kidney,* A. manshuriensis* has been banned in China. Thus, although some alternatives including* Akebia trifoliata* (Thunb.) Koidz and* Akebia trifoliata* (Thunb.) Koidz. var.* australis* (Diels) Rehd have been applied, the supply of* Akebia caulis* is still scarce. In the current market of Chinese Materia Medica, the price of* Akebia caulis* is about 30–50 RMB/kg, which is obviously higher than a decade ago and still rising. In this situation, the discovery of a new source becomes particularly urgent.

*Stauntonia brachyanthera* Hand-Mazz., an evergreen shrub, is naturally growing in the southwest of China including Hunan, Guizhou, and Guangxi provinces. Just as* A. trifoliata* or* A. trifoliate*,* S. brachyanthera* also belongs to the family of Lardizabalaceae. In local areas, this plant is a main economic crop, whose fruits are not only consumed for its delicious taste, but also used as raw materials to produce beverages and vinegar [13]. Meanwhile, its seeds are utilized to extracting oil for edible purposes. In addition,* S. brachyanthera* also has its medicinal values. In Dong and Yao minorities of China, it has been used as the alternative of* Akebia caulis* for the purpose of diuresis and the treatments of inflammation, pain, and edema [14]. Besides, the constituents from* S. brachyanthera* are also proved to have antioxidant, cytotoxic [15], and hepatic protectant activities [16].

From the aspect of medicines,* S. brachyanthera* has not only the same origin as other plants of* Akebia caulis*, but also similar pharmacological effect, which has been applied in some regions. So,* S. brachyanthera* is probably the succedaneum and new resource of* Akebia caulis*. It is interesting to note that in local countryside of the southwest of China, farmers usually use the stem of* S. brachyanthera* as pipe to blow toward the fire to make the flame more exuberant when cooking. This utilization is just the folk usage of* Akebia caulis* and the origin of the name of* Akebia caulis* (Mutong, in China). So, it could be presumed that the physiologic structures of* S. brachyanthera* and* Akebia caulis* might be much alike. Therefore, in order to scientifically verify this thesis and find more effective diuretics, a serious of researches including the microscopic features, the diuretic effect and the analysis and identification of main constituents of* S. brachyanthera* were carried out. Herein, the details of these works will be discussed comprehensively.

## 2. Materials and Methods

### 2.1. Plant Material

Samples of* S*.* brachyanthera* were collected in Hunan by Shumo Mei, Huaihua Medical College in October 2009, and were identified by Professor Jincai Lu, School of Traditional Chinese Materia Medica, Shenyang Pharmaceutical University. A voucher specimen (number HLG-0910) was deposited in the School of Traditional Chinese Material Medica, Shenyang Pharmaceutical University.

### 2.2. The Preparation of Microtome Section

Dried stems of plants were boiled for 3 hours in water and then cut into 20 *μ*m of slices. After dyeing with sarranine and red orange, the samples were mounted for further observation.

### 2.3. Animals and Drugs

Male albino rats of the Wistar strain, weighing approximately 220–280 g, obtained from the Experimental Animal Center of Shenyang Pharmaceutical University, Shenyang, China, were acclimatized under laboratory conditions with free access to commercial chow and water for 2 weeks. Wistar rats under* ad libitum* water conditions and fasted for 18 h prior to the start of the selection were administered with deionized water (2.5% body wt, i.g.) and their urine volume were measured after 2 h following the method of Aston [17]. Only those whose urinary outputs were more than 1% body wt can be used for the experiment.

Animals and all experiments were performed according to the approved protocols of Animal Ethics Committee, Shenyang Pharmaceutical University, China (SCXK (Liao) 2010-0001).

Furosemide was purchased from Tasly Company (Tianjin, China). 0.9% normal saline was purchased from Dubang Company (Jilin, China). Furosemide and the extracts of* S. brachyanthera* were dissolved in normal saline.

### 2.4. Diuresis Study

In the diuretic experiments, animals were randomly divided into 5 groups of eight animals. The normal control group received vehicle only and the furosemide-treated groups were given furosemide (10 mg/kg body wt, i.g.). The treatment groups were intragastrically administered ESB at doses of 150, 300, and 600 mg/kg, respectively.

Wistar rats under* ad libitum* water conditions and fasted for 12 h prior to the start of the experiment were volume-expanded with 0.9% NaCl (4% body wt, i.g.) vehicle, and furosemide and different doses of ESB were administered to each group of rats, respectively, as indicated in the text. Rats were individually housed in metabolic cages, and urine volume was measured every 60 min throughout the experiment (6 h).

### 2.5. Extraction and Isolation of* S. brachyanthera*

The stems of* S. brachyanthera* were chopped into small pieces (200 mesh) and then extracted with 70% aqueous EtOH under reflux for 2 h. After evaporation of the combined EtOH extracts* in vacuo*, the resultant aqueous residues were heated into dryness to get the extracts (ESB). The 2.0 kg of ESB was suspended in water and passed through macroporous adsorptive resin (HPD-100, Cangzhou Bon Adsorber Technology Co., Ltd., Cangzhou, China) and eluted sequentially with H_2_O, 40% EtOH, and 95% EtOH to afford 40% EtOH eluates (MR40) and 95% EtOH eluates (MR95), respectively.

MR40 (180 g) was chromatographed on silica gel (200–300 mesh, Qingdao Haiyang Chemical Group Corporation, Qingdao, China) column with a gradient CH_2_Cl_2_-MeOH system (100 : 1–0 : 100) to give nine fractions (Fr. 40**A**–40**I**). Those fractions were further purified by repeated silica gel column chromatography (CC) with PE/EtOAc and CHCl_3_/MeOH as eluants, Sephadex LH-20 (GE Healthcare, Uppsala, Sweden) CC with MeOH as mobile phase, ODS CC (YMC-Pack-ODS, 50 *μ*m, YMC Co. Ltd., Kyoto, Japan) eluted by MeOH/H_2_O (10 : 100–100 : 80), and preparative reverse phase high pressure liquid chromatography (RP-HPLC, YMC-Pack ODS-A, 250 × 20 mm, 5 *μ*m) to give** 4 **(24.7 mg) from fraction** C**,** 12** (380 mg) and** 18** (12.0 mg) from fraction** D**,** 2** (25.0 mg),** 3** (16.4 mg),** 19** (14.9 mg), and** 20** (21.2 mg) from fraction** E**,** 8** (12.0 mg) and** 7 **(36.6 mg) from fraction** G**,** 16** (962.5 mg) and** 21** (2.1 g) from fraction** H**, and** 1** (586.8 mg),** 9** (23.0 mg), and** 10** (1.2 g) from fraction** I**, respectively. MR95 (65 g) was also chromatographed on silica gel column with a gradient CH_2_Cl_2_-MeOH system (100 : 1–0 : 100) to give 6 fractions (Fr. 95**A**–95**F**). Those fractions were further purified by repeated silica gel CC with PE/EtOAc and CHCl_3_/MeOH as eluants, Sephadex LH-20 CC with MeOH as mobile phase, and recrystallization method to give compounds** 5 **(15.3 mg) and** 6 **(61.2 mg) from fraction** B**,** 13 **(24.7 mg) and** 17** (36.6 mg) from fraction** C**,** 11 **(20.1 mg) from fraction** E**,** 15** (12.0 mg) from fraction** H**, and** 22** (23.0 mg),** 14** (16.8 mg), and** 23** (32.4 mg) from fraction** I**, respectively. All chemicals and solvents used in this study were of analytical grade.

The structures of all isolated compounds were determined by NMR spectra, which were acquired using a Bruker ARX-300 and ARX-600. Chemical shifts (*δ* ppm) were relative to TMS as an internal standard.

### 2.6. Statistical Analysis

Values were expressed as means ± SEM. The significance of differences between means was analyzed by repeated-measure analysis of variance (ANOVA). A *p* value less than 0.05 was considered significant.

## 3. Results

### 3.1. Pharmacognosy Characteristics

**Figure 1:**
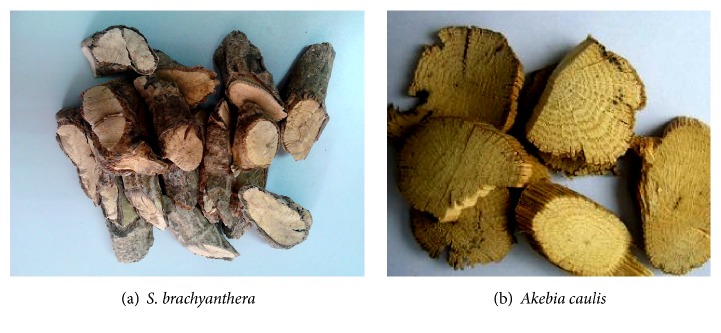
The crude drugs of the stems of* S. brachyanthera* and* Akebia caulis*.

The stem of* S. brachyanthera* (Figure 1(a)) was cylindrical with the diameter of 1~3 cm or more thick. Its surface is rough and wrinkled with many irregular furrows and lenticel-like protrusions on it. The plant is light, while the texture is compact and tough. The cortex of the stem was thick and in the color of yellowish-brown or greyish-yellow. The inner wood was also yellowish-white with small port-holes, which ensured the traverse of the air. The yellowish-brown rays were arranged radially and the pith was also yellowish-brown and small. All of these characteristics of the appearance of the stem of* S. brachyanthera* were much alike to that of* Akebia caulis* according to our observation (Figure 1(b)) and the description in Chinese Pharmacopeia [12].

**Figure 2:**
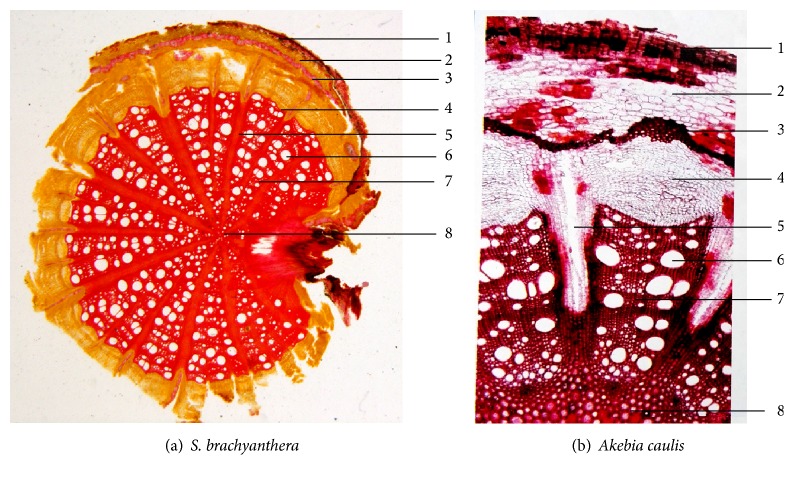
Microscopic features of transverse section of stems of* S. brachyanthera* and* Akebia caulis*. 1: cork, 2: cortex, 3: stone cells, 4: phloem, 5: ray, 6: vessel, 7: xylem, and 8: cord.

Observed from the microscope, it could be found that microscopic features of the two kinds of medicinal materials were almost the same. In the transverse section of the stem of* S. brachyanthera* (Figure 2(a)), the thick cork consisted of several layers of cells which filled with yellowish-brown contents. The phellogen consisted of 2~3 rows of tangentially elongated cells. The phelloderm was comprised of several layers of flat cells with intermittent circular belt of rounded stone cells inside. The cortex was composed of rounded parenchyma cells which were filled with yellowish-brown contents. The vascular cylinder was broad, scattered with ectophloic vascular bundle and with wavy pericyclic fiber outside. In the transverse section, it could also be found that the phloem of the stem of* S. brachyanthera* was narrow, while the xylem was broad. The interfascicular cambium was indistinct and irregular shape stone cells could be observed in the outer of interfascicular cambium. The cells in the pith were thick-walled and lignified.

The similar microscopic features could also be found in the transverse section of* Akebia caulis* (Figure 2(b)), in which there were several rows of cork cells containing brown contents. The phelloderm was filled with plenty of calcium oxalate prisms. The cortex of* Akebia caulis* was composed of 6~10 rows of parenchyma cells which were filled with small prisms too. The stone cells observed from microscope were also arranged in a circular belt. The phloem was narrow. The xylem was composed of vessel, wood fiber, and parenchyma cells, whose cell walls were all lignified. And the rays of* Akebia caulis* were all primary rays [18].

### 3.2. Diuretic Effect of ESB on Rats

In order to evaluate acute alterations in diuresis, a 4% body weight volume expansion was produced in all experiments. This magnitude of expansion allowed studies to be performed on the acute effect of substances that either stimulate or inhibit diuresis [19].

**Figure 3:**
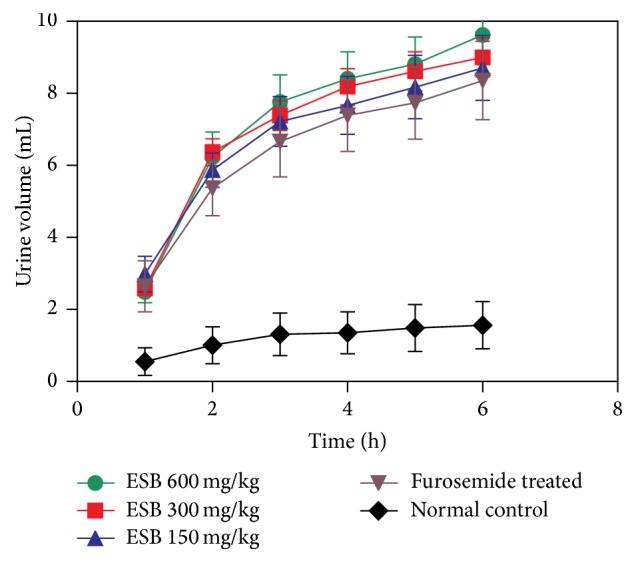
The diuretic effects of ESB on rats.

From the results of diuretic effects as shown in Table 1 and Figure 3, it could be found that, in the experimental conditions, furosemide (10 mg/kg) could increase the urine volume significantly compared with normal control. For example, furosemide increased urine volume from 0.55 ± 0.38 mL (normal control) to 2.64 ± 0.71 mL (*p* < 0.01) at the first hour and reached to 7.73 ± 1.00 mL (*p* < 0.001) at 5th hour compared with 1.49 ± 0.65 mL of normal control. ESB also showed similar effect as furosemide at a low dose (150 mg/kg), whose accumulated urine volume was 2.98 ± 0.49 mL (*p* < 0.001) at first hour and 8.17 ± 0.88 mL (*p* < 0.001) at 5th hour. Compared with furosemide, the diuretic effects of ESB at middle and higher doses exhibited relative stronger effects. From 2nd hour to 6th hour, for example, the accumulated urine volumes of ESB 300 mg/kg treatment group were all higher than those of furosemide treatment group, and ESB 600 mg/kg could stimulate rats' urine production at the highest level, which could be observed directly from Figure 3.

From Figure 3, it also could be found that both ESB and the furosemide could cause the increase of urine volume at the same manner. They stimulated the urine volume of rats very obviously during the first four hours, especially the first two hours. For example, the accumulated urine volume of ESB at 300 mg/kg dose was 6.36 ± 0.37 mL (*p* < 0.001) at the first hour and then reached to 8.18 ± 0.51 mL (*p* < 0.001) at the 4th hour. But from 4th hour to 5th hour, it only increased the urine volumes from and 8.18 ± 0.51 mL (*p* < 0.001) to 8.61 ± 0.54 mL (*p* < 0.001). After four hours later, the degree of the increase of urine volumes was lowered. From this result, it can be drawn that the optimal time for ESB to take effect should be the first four hours, especially the first two hours.

Although ESB could stimulate the increase of rat's urine volume at both 300 and 600 mg/kg doses, there were no obvious differences between two doses. So, from the aspect of convenience of drug administration, the optimum dose of ESB for its diuretic action should be at the 300 mg/kg body wt. In addition, it must be pointed out that ESB could continuously play pharmacological effects at all three doses after six hours later, which indicated its longer period of effecting time for the diuretic properties.

### 3.3. Structural Elucidation of the Chemical Constituents from* S. brachyanthera*

**Figure 4:**
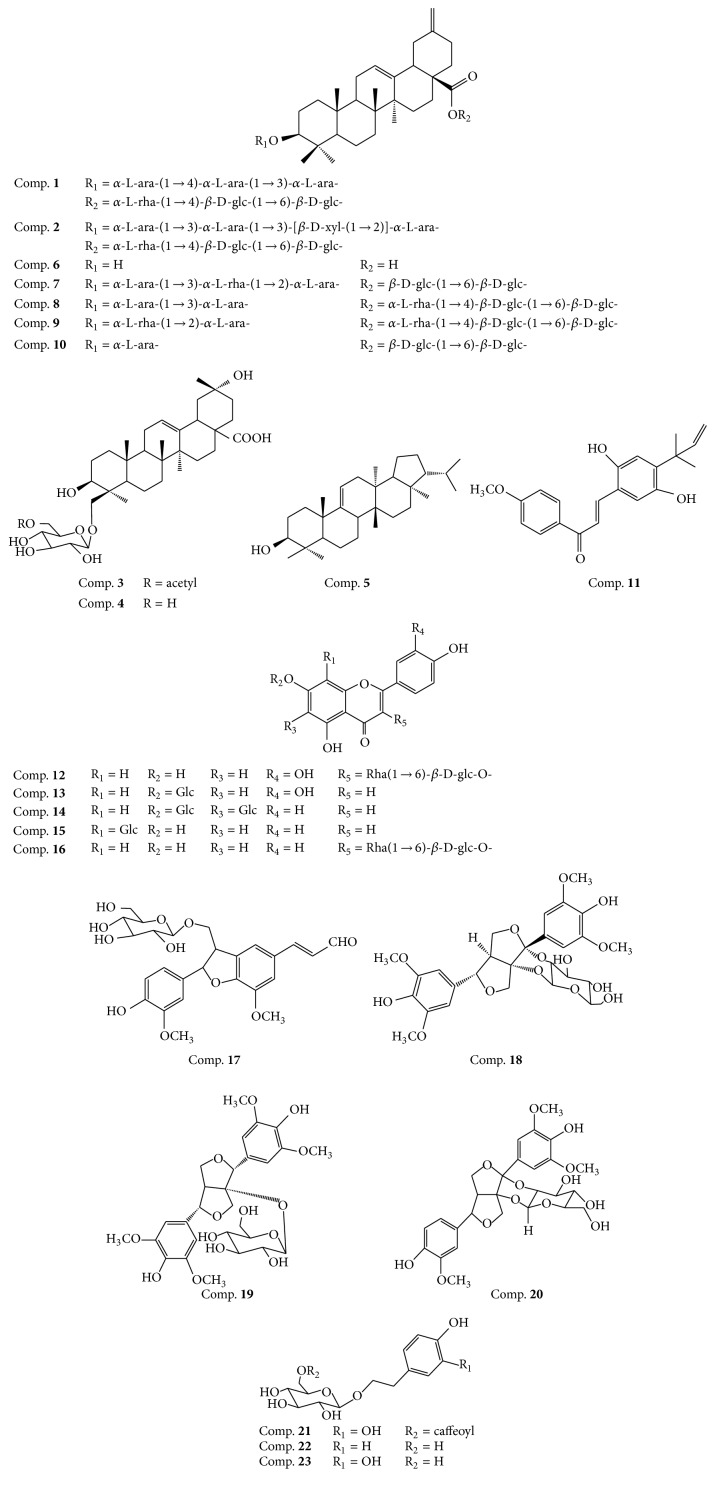
The structures of isolated compounds from 70% EtOH extracts of* S. brachyanthera*.

The detail studies on ESB by various chromatographic methods finally lead to the isolation of 23 compounds. By comparing their ^1^H and ^13^C NMR data with reported values, the structures of these compounds were identified as brachyantheraoside A_4_ (**1**), brachyantheraoside A_5_ (**2**), brachyantheraoside B_6_ (**3**) [19], 3*β*, 20*α*, 24-trihydroxy-29-norolean-12-en-28-oicacid 24-*O*-*β*-D-glucopyranoside (**4**) [20], fernenol (**5**) [21], 3*β*-3-hydroxy-30-norolean-12, 20(29)-dien-28-oic acid (**6**) [22], yemuoside I (7) [23], yemuoside YM_7_ (**8**), yemuoside YM_10_ (**9**), yemuoside YM_11_ (**10**), licochalcone A (**11**) [24], rutin (**12**) [25], luteolin-7-O-glucoside (**13**) [26], saponarin (**14**) [27], vitexin (**15**) [28], kaempferol-3-O-rutinose (**16**) [29], tortoside F (**17**) [30], brachyanin E (**18**) [15], staunoside C (**19**) [31], 7-(4-hydroxy-3-methoxyphenyl)-7′-(4′-hydroxy-3′,5′-dimethoxyphenyl)-7,9′:7′,9-diepoxylignan-8H,8′-*O*-*β*-D-(2′′,7′-epoxy)-glucopyranoside (**20**) [32], calceolarioside B (**21**) [33], 2-(4′-hydroxyphenyl)ethyl-*β*-D-glucopyranoside (**22**) [34], and 2-(3′,4′-dihydroxyphenyl)ethyl-*β*-D-glucopyranoside (**23**) [35]. Among them, compounds** 5**,** 6**,** 7**,** 11**,** 12**,** 16**, and** 17** were firstly reported in the genus of* Stauntonia*, which not only was a breakthrough on the study of* S. brachyanthera*, but enriched the structural types of the genus. Their structures were listed in Figure 4.

## 4. Discussion and Conclusion

In this paper, by the comparative observation of pharmacognosy characteristics of the fork medicine,* S. brachyanthera* (Figures 1(a) and 2(a)), and the traditional Chinese medicine,* Akebia caulis* (Figures 1(b) and 2(b)), it could be found that the characters of crude drugs of the stem of* S. brachyanthera* are similar to the properties of* Akebia caulis*. The transverse sections of two plants were all presented in the form of spider's web, which marks Mutong off from other medicinal materials. As observed from microscope, it could be found that the ratios of xylem in both medicinal materials were significantly higher than the other parts, while those of core were little. Meanwhile in the xylem, there were abundant of vessels, whose diameters were obviously big. Another important feature in common between two medicinal materials was that there were all a distinct layer of stone cells filled with prisms of calcium oxalate in phelloderm, which was usually the main evidence for the identification of medicinal materials. Therefore, as concluded, all those common characteristics, including the spider's web transverse section, plentiful vessels in xylem, and stone cells circular band, indicated that* S. brachyanthera* were almost similar to* Akebia caulis* and might be used as the substitution of* Akebia caulis* from the point of pharmacognosy.

Just as discussed above,* Akebia caulis* has been used in China as diuretics for a long time and its effect diuresis has also been proved [36–38]. In our present studies, the diuretic studies also showed that the ethanol extracts of the stems of* S. brachyanthera* have significant effects on diuresis in normal rats. At low dosage (150 mg/kg body wt), the effects of ESB are consistent with the furosemide treatment group. While the outputs of urine of ESB at middle and high dosages (300 mg/kg, 600 mg/kg body wt) were obviously increased compared to that of furosemide treatment group, the trends of later four hours were similar to the furosemide treatment group. Therefore the results of pharmacological study* in vivo* demonstrated that ESB have strong diuretic effect, and the optimum time for ESB to take effects is the first four hours, especially the first two hours, which could reveal that* S. brachyanthera* might be a succedaneum of the* Akebia caulis* from the level of pharmacology.

The chemical studies on ESB led to the isolation of a variety of compounds including triterpenoids (Comp.** 1**–**10**), flavonoids (Comp.** 10**–**16**), lignanoids (Comp.** 17**–**20**), and phenylethanoid glycosides (Comp.** 21**–**23**). The types of these compounds were much similar to those of* A. quinata* and consistent with those of the plants in Lardizabalaceae family [14]. Among them, the amounts of brachyantheraoside A_4_ **1** (586.8 mg), yemuoside YM_11_ **10** (1.2 g), rutin** 12** (380 mg), kaempferol-3-O-rutinose** 16** (962.5 mg), and calceolarioside B** 21** (2.1 g) were significantly higher than the other compounds, which could be designated as characteristic components for the qualitative identification of the plant. Calceolarioside B, the characteristic component of* Akebia caulis* in Chinese Pharmacopeia [12], could also be served as indicator because of its absolutely higher amount for the quality control of the* S. brachyanthera*. Thus, from the chemical aspect including the structural types and the amount of calceolarioside B, it could be deduced that* S. brachyanthera* has the similar chemical composition and might be applied as the succedaneum of* Akebia caulis*.

*Akebia caulis* and* S. brachyanthera* come from different genus of the same family. Their consistent pharmacognosy characteristics and similar chemical constituents further confirmed the close genetic relationship between them and revealed the similar pharmacodynamic materials and the same pharmacological effect. Our study presented sufficient evidences for the substitution of* S. brachyanthera* for* Akebia caulis*, which will probably expand the resources of* Akebia caulis*, solve the supply shortage, and promote the full utilization of this economic crop.

As for the diuretic effect,* S. brachyanthera* can be considered as a promising plant for further studies and applications in the production of bioactive ingredients, not only in food or functional food industry, but also in pharmaceutical industry. Thus, more comprehensive studies should be focused on the diuretic effects of the main composition such as yemuoside YM_11_, kaempferol-3-O-rutinose, or calceolarioside B by* in vivo* and* in vitro* experiments, which are undergoing in our group and will be reported later.

## Figures and Tables

**Table 1:** The accumulated urine volumes of rats by treating different doses of ESB.

Substance (mg/kg)	Accumulated urine volume (mL)					
	+1 h	+2 h	+3 h	+4 h	+5 h	+6 h
None	0.55 ± 0.38	1.01 ± 0.51	1.36 ± 0.59	1.35 ± 0.58	1.49 ± 0.65	1.56 ± 0.66
ESB 150	2.98 ± 0.49^*∗*^	5.87 ± 0.48^*∗*^	7.22 ± 0.69^*∗*^	7.66 ± 0.80^*∗*^	8.17 ± 0.88^*∗*^	8.70 ± 0.90^*∗*^
ESB 300	2.59 ± 0.04^#^	6.36 ± 0.37^*∗*^	7.38 ± 0.41^*∗*^	8.18 ± 0.51^*∗*^	8.61 ± 0.54^*∗*^	8.99 ± 0.54^*∗*^
ESB 600	2.49 ± 0.30^#^	6.23 ± 0.70^*∗*^	7.76 ± 0.75^*∗*^	8.41 ± 0.75^*∗*^	8.82 ± 0.74^*∗*^	9.62 ± 0.74^*∗*^
Furosemide	2.64 ± 0.71^#^	5.37 ± 0.77^*∗*^	6.66 ± 0.98^*∗*^	7.39 ± 1.00^*∗*^	7.73 ± 1.00^*∗*^	8.36 ± 1.09^*∗*^

Data are represented as means ± SEM; number in parenthesis is the number of rats used; nd: not determined; ^#^ *p* < 0.01 and ^*∗*^ *p* < 0.001  *versus* control (none).
